# Eosinophils in Chronic Urticaria: Supporting or Leading Actors?

**DOI:** 10.1097/WOX.0b013e3181bb965f

**Published:** 2009-09-15

**Authors:** Riccardo Asero, Massimo Cugno, Alberto Tedeschi

**Affiliations:** 1 Allergy Unit, Clinica San Carlo, Paderno Dugnano (Milan), Italy; 2 Department of Internal Medicine, University of Milan and IRCCS Foundation Maggiore Hospital, Mangiagalli and Regina Elena, Milan, Italy; 3 Allergy and Clinical Immunology Unit, IRCCS Foundation Maggiore Hospital, Mangiagalli and Regina Elena, Milan, Italy

**Keywords:** chronic urticaria, mastcells, eosinophils, VEGF, tissue factor

## Abstract

Although their number may be increased in skin lesions, eosinophils have been rather neglected as possible participants to the pathogenesis of chronic urticaria because of the absence of peripheral eosinophilia in patients with this disease. However, recent data suggest a potentially relevant role played by activated eosinophils both in triggering the tissue factor pathway of coagulation cascade and as a source of vascular endothelial growth factor. Such phenomena seem more pronounced in patients showing a more severe disease. The present study will rediscuss the potential role of this cell line in chronic urticaria in the light of these recent observations.

## Introduction

Chronic urticaria (CU), defined as the occurrence of spontaneous wheals, with or without angioedema, for more than 6 weeks is a rather frequent disorder. As in most other types of urticaria, the pathophysiologic basis of CU is unquestionably the recurrent degranulation of dermal mast cells and of basophils. Mast cells and, to a lesser extent, basophils, are generally regarded as the main effectors of this disease. However, the events occurring upstream and eventually resulting in histamine release from these cells are partially defined. Studies carried out during the last 2 decades have led to the detection of functional autoantibodies to the high affinity IgE receptor, Fc*ε*RI, or to IgE that are able to cause degranulation and histamine release from both mast cells and basophils [1-4]. Complement plays a relevant role in this process by enhancing histamine release induced by Fc*ε*RI autoantibodies [5,6]. However, it is generally agreed that autoantibodies to Fc*ε*RI or to IgE can be detected in less than 50% of CU patients. Furthermore, there is increasing evidence that autoantibodies and in vivo autoreactivity can also be detected in other conditions and in healthy subjects [7-10]. All this means that the factors triggering histamine release in chronic urticaria remain a mystery in many cases. The reported efficacy of omalizumab in patients with refractory CU either with or without detectable autoantibodies [11-15] and the observed, although not totally specific, inhibitory effect of heparin on autoantibody-triggered histamine release from basophils in vitro,[16,17] suggest a common final pathway in histamine release, irrespective of the histamine-releasing factor involved.

## Histology of CU

It is generally accepted that the histologic picture is more or less the same in all patients with CU irrespective of the presence or absence of autoantibodies. A perivascular infiltrate of CD4^+ ^lymphocytes is always present [18]. Infiltrating cells have the characteristics of both T_H_1 and T_H_2 cells; increased numbers of intradermal CD3, CD4, CD8-positive T cells have been detected with a T_H_0 cytokine profile. Neutrophils and a variable degree of eosinophils are also present [19,20]. Eosinophils are often activated, particularly in patients without autoantibodies,[20] and major basic protein can be measured in urticaria lesion even when eosinophils are not detected [21]. The number of mast cells has been reported as increased in urticaria lesions [18] although this finding has not been confirmed by more recent studies,[19,22] and some basophil infiltration is also observed. It has been suggested that the infiltrate is similar to that of an allergy late-phase reaction [23,24].

**Figure 1:**
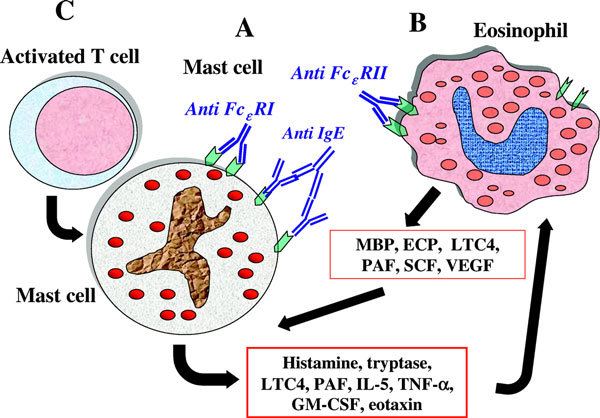
**Immune mechanisms and main effector cells involved in chronic urticaria**. A, Mast cells are activated either by autoantibodies to Fe*ε*RI or IgE and/or by other histamine releasing factors and release several mediators (histamine, leukotrienes, VEGF) that concur to produce the marked vasodilation that stands at the basis of both wheal-and flare reaction and angioedema. Some mediators and chemokines released by mast cells can recruit and activate eosinophils that in turn release inflammatory mediators and produce tissue factor, the main initiator of the extrinsic pathway of the coagulation cascade. The major basic protein released by eosinophils can induce mast cell degranulation. B, Eosinophils can be activated either directly by autoantibodies against the low affinity IgE receptor or indirectly by mast-cell derived mediators. C, Activated T-cells can induce mast cell degranulation by cell-to-cell contact. This process leads to the formation and release of cytokines such as TNF-*α *that has the capacity to induce gene expression in mast cells by an autocrine mechanism. ECP, eosinophil cationic protein; GM-CSF, granulocyte-monocyte colony-stimulating factor; MBP, major basic protein; PAF, platelet-activating factor; SCF, stem cell factor.

Interestingly, the eosinophil-derived major basic protein has been identified in autologous serum skin test site biopsies, along with eosinophil infiltration [25]. The immune mechanisms and the main effector cells involved in chronic urticaria are shown in Figure 1.

## Who Activates Mast Cells When Autoantibodies are not Present?

During the last 5 years, our group has spent some time and effort trying to respond to this question by measuring the circulating levels of some mediators potentially able to induce mast-cell degranulation and histamine release. In view of the constant absence of blood eosinophilia in CU, we measured serum levels of some substances not specifically related with eosinophils, namely stem cell factor,[26] substance P,[27] and interleukin (IL)-18 [28]. However, the concentration of these mediators did not significantly differ between patients and controls. Recently, we have been luckier when, based on the observation of a higher prevalence of positive autologous plasma skin test over autologous serum skin test, we started looking at the coagulation cascade and were able to demonstrate that CU is characterized by thrombin generation,[29] which is the result of an activation of the tissue pathway of blood coagulation [30,31]. Notably, studies in animal models have shown that thrombin is able to induce mastcell degranulation,[32] and in rat mast cell populations, the response to thrombin is equipotent with Fc*ε*RI-mediated activation [33]. Thus (although evidence that thrombin induces mast cells degranulation in humans is still missing), thrombin might be a candidate mast-cell activating factor, at least in patients whose sera don't contain autoantibodies and show an activation of coagulation cascade. One good question is if thrombin activates all mast cells, why do we see just skin mast cell activation in CU? In other words, why is there skin selectivity in CU? In effect, there are studies showing that in CU patients there is bronchial hyper-responsiveness and, sometimes, frank asthma [34,35].

## Eosinophils as Activators of the Coagulation Cascade and as Source of Vegf in Chronic Urticaria (Figure 2)

Interestingly, although the role of other cells cannot be ruled out, investigating further the activation of the coagulation cascade, we found that the cells expressing tissue factor in chronic urticaria, and hence triggering the activation of the extrinsic pathway, are eosinophils [36]. In fact, immunohistochemical experiments showed that tissue factor colocalized with eosinophil cationic protein, a classic cell marker of eosinophils. These findings highlight the importance of eosinophils in chronic urticaria as a source of tissue factor, in keeping with recent studies showing that eosinophils store tissue factor and rapidly transfer it to the cell membrane during activation [37]. The strong expression of tissue factor in chronic urticaria lesional skin may be because of eosinophil activation, even if patients with chronic urticaria do not show peripheral eosinophilia, probably because tissue factor facilitates the early transendothelial migration of the eosinophils [37]. It is interesting to note that eosinophils in chronic urticaria may be activated both by direct and indirect mechanisms. In a single, hitherto unconfirmed, study autoantibodies directed against CD23, the low-affinity IgE receptor located on eosinophil membrane, have been detected in about 65% of chronic urticaria patients. These autoantibodies can activate eosinophils inducing the release of major basic protein, which in turn causes mast cell degranulation [38]. In addition, eosinophil involvement might be secondary to the mast cell activation caused by anti-Fc*ε*RI and anti-IgE autoantibodies or other histamine-releasing factors. It has been demonstrated that several mediators and cytokines released by activated mast cells can recruit and stimulate eosinophils. Among others, mast cells represent an important source of IL-5, tumor necrosis factor (TNF)-*α, *platelet-activating factor and eotaxin, molecules that can exert potent chemotactic and stimulating activities on eosinophils [39,40].

**Figure 2:**
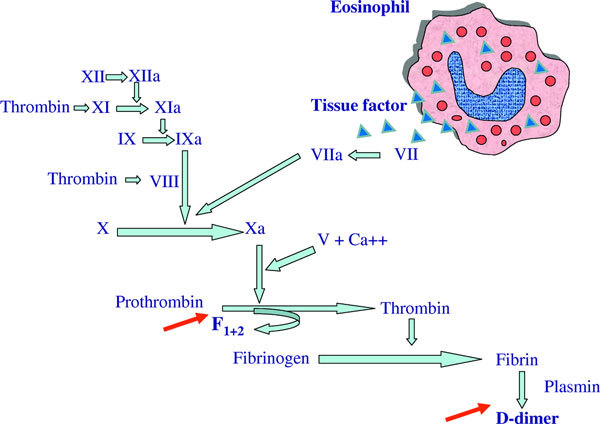
**Activation of the extrinsic pathway of the coagulation cascade by eosinophil-derived tissue factor in patients with chronic urticaria**. Eosinophils produce and store tissue factor (blue triangles), and rapidly transfer it to the cell membrane during activation. Activation of the coagulation cascade and fibrinolysis in patients with chronic urticaria is demonstrated by the increased plasma levels of the prothrombin fragment F1+2 and D-dimer (indicated by red arrows).

One further recent observation is that patients with CU show a significant increase of vascular endothelial growth factor (VEGF) [41]. VEGF is the most potent regulator of angiogenesis presently known, and one of the major mediators of vascular permeability; furthermore, it exerts a vasodilator effect through an increase of nitric oxide production by endothelial cells [42,43]. Notably, eosinophils are the main source of VEGF in CU as well,[41] in agreement with the in vitro observations that human peripheral blood eosinophils have the capacity to induce angiogenesis [44]. All these findings clearly suggest that eosinophils might play a role in chronic urticaria.

## Who is Activating Whom?

As reviewed above, it seems rather clear that both mast cells and eosinophils are activated in CU. Who is the one that starts all the process? Is eosinophil activation the result of mast cell-derived mediators release or are eosinophils themselves the cause of mast cell activation and subsequent histamine release?

It cannot be excluded that the leading actor may change in different subsets of patients. In patients showing circulating Fc*ε*RI and anti-IgE autoantibodies it is very likely that the main actor is the mast cell whereas in other patients showing only anti-Fc*ε*RII autoantibodies such role might be played by eosinophils. Finally, it cannot be excluded that T cells, which have been found in chronic urticaria lesional skin, also play a relevant role, possibly by activating the mast cells via a cell-to-cell contact. In fact, it has been demonstrated that T cells can activate mast cells by cell-to-cell contact, thus provoking the release of cytokines and inflammatory mediators such as histamine, TNF-*α *and matrix metalloproteinase 9 [45,46]. Furthermore, an aberrant signaling through the p21Ras pathway has been found in peripheral blood mono-nuclear cells of patients with chronic urticaria, a finding that is in agreement with lymphocyte activation and supports the autoimmune basis of the disease [47]. Finally, the clinical response of chronic urticaria to corticosteroid treatment suggests to the importance of the cellular infiltrate (T cells, eosinophils, or even basophils) because mast cells tend to be steroid-resistant.
